# Independent or simultaneous lowering of core and skin temperature has no impact on self-paced intermittent running performance in hot conditions

**DOI:** 10.1007/s00421-019-04173-y

**Published:** 2019-06-20

**Authors:** G. Thomas, T. Cullen, M. Davies, C. Hetherton, B. Duncan, N. Gerrett

**Affiliations:** 1 School of Sport and Exercise Science, University of Worcester, Worcester, UK; 2 Faculty of Human Movement Sciences, Vrije Universiteit Amsterdam, Amsterdam, The Netherlands; 3 Centre for Sport Exercise and Life Sciences, Coventry University, Coventry, UK; 4 Laboratory for Applied Human Physiology, Graduate School of Human Development and Environment, Kobe University, Kobe, Japan

**Keywords:** Thermoregulation, Performance, Team sports, Self-pacing, Pre-cooling, Intermittent exercise

## Abstract

**Purpose:**

To investigate the effects of lowering core (*T*_gi_) and mean skin temperature (*T*_sk_) concomitantly and independently on self-paced intermittent running in the heat.

**Methods:**

10 males (30.5 ± 5.8 years, 73.2 ± 14.5 kg, 176.9 ± 8.0 cm, 56.2 ± 6.6 ml/kg/min) completed four randomised 46-min self-paced intermittent protocols on a non-motorised treadmill in 34.4 ± 1.4 °C, 36.3 ± 4.6% relative humidity. 30-min prior to exercise, participants were cooled via either ice slurry ingestion (INT); a cooling garment (EXT); mixed-cooling (ice slurry and cooling garment concurrently) (MIX); or no-cooling (CON).

**Results:**

At the end of pre-cooling and the start of exercise *T*_gi_ were lower during MIX (36.11 ± 1.3 °C) compared to CON (37.6 ± 0.5 °C) and EXT (36.9 ± 0.5 °C, *p* < 0.05). Throughout pre-cooling *T*_sk_ and thermal sensation were lower in MIX compared to CON and INT, but not EXT (*p* < 0.05). The reductions in thermophysiological responses diminished within 10–20 min of exercise. Despite lowering *T*_gi_, *T*_sk_, body temperature (*T*_b_), and thermal sensation prior to exercise, the distances covered were similar (CON: 6.69 ± 1.08 km, INT: 6.96 ± 0.81 km, EXT: 6.76 ± 0.65 km, MIX 6.87 ± 0.70 km) (*p* > 0.05). Peak sprint speeds were also similar between conditions (CON: 25.6 ± 4.48 km/h, INT: 25.4 ± 3.6 km/h, EXT: 26.0 ± 4.94 km/h, MIX: 25.6 ± 3.58 km/h) (*p* > 0.05). Blood lactate, heart rate and RPE were similar between conditions (*p* > 0.05).

**Conclusion:**

Lowering *T*_gi_ and *T*_sk_ prior to self-paced intermittent exercise did not improve sprint, or submaximal running performance.

**Electronic supplementary material:**

The online version of this article (10.1007/s00421-019-04173-y) contains supplementary material, which is available to authorized users.

## Introduction

Training and competition for many intermittent team sports take place in environmental conditions exceeding 30 °C, as seen during the 2014 Fédération Internationale de Football Association (FIFA) World Cup in Brazil (Nassis et al. 2015). During a soccer game in 43 °C and 12% relative humidity (RH), a 7% decline in total game distance covered and a 7% reduction in high-intensity running was reported compared to cooler conditions (21 °C, 55% RH) (Mohr et al. 2012). Cooling administered before intermittent exercise in the heat may have a potential benefit to mitigate the reduction in performance (*d* = 0.47) (Tyler et al. 2015), but the effectiveness of pre-cooling appears to be dependent on the magnitude of thermal strain experienced and the volume of the cooling applied. Recently, we demonstrated that pre-cooling via ice slurry ingestion (7.5 g/kg) successfully lowered core temperature and thermal sensation with no change in mean skin temperature (*T*_sk_)_,_ in comparison to a control beverage (Gerrett et al. 2017). However, lowering core temperature alone did not result in favourable changes to intermittent exercise performed in 30 °C, 40% RH. Studies demonstrating some evidence of enhanced intermittent exercise following pre-cooling report a decrease in both core temperature and *T*_sk_ (Duffield and Marino 2007; Minett et al. 2011). Thus, supporting the notion of a dose-dependent response to precooling; the larger the surface exposed to cooling the greater the distance achieved during submaximal running.

Despite mixed method pre-cooling offering more cooling power compared to singular use (Bongers et al. 2015), research investigating external and internal mixed method cooling on subsequent intermittent running performance is limited and conflicting. Effective strategies and appropriate mechanisms need elucidating. A recent meta-analysis could not demonstrate a statistically significant (*p* = 0.28) overall weighted effect of mixed-method pre-cooling on intermittent performance (Hohenauer et al. 2018). Aldous et al. (2018) investigated the use of mixed-method pre-cooling 30-min prior to a 45-min intermittent protocol in the heat (30.7 °C, RH 50.9%). Total distance, high-speed distance and variable run distance were significantly improved in comparison to a control condition (Aldous et al. 2018). Whereas Brade et al. (2013), in a group of acclimatised males, found repeated sprint running performance was not improved following a 30-min mixed-method pre-cooling protocol [ice slurry (7 g/kg) and cooling jacket]. This may have been attributed to only moderate reductions in core temperature (− 0.4 °C) and no differences in *T*_sk_ compared to a control condition. Thermal sensation, an important behavioural thermoregulatory controller of exercise intensity and performance may be more favourable when both core temperature and *T*_sk_ are lowered (Faulkner et al. 2015; Sawka et al. 2011; Schlader et al. 2011). However, it is important to note, Brade et al. (2013) and some of the studies included in the Hohenauer et al. (2018) meta-analysis used participants already acclimatised prior to mixed-method pre-cooling. Thus, evidence suggests mixed-method pre-cooling maybe unnecessary if seasonally acclimatized or heat-acclimated (Brade et al. 2013; Castle et al. 2011).

To date, results examining the performance effect of mixed-method pre-cooling on intermittent running exercise in the heat is equivocal. More research is warranted to gain a clear understanding of the benefit of using a mixed-method pre-cooling protocol to investigate the role of lowering core temperature and *T*_sk_ on intermittent exercise performance. Given that team sports are inherently self-paced, non-motorised treadmills (NMT) have allowed the development of internally paced performance tests, offering a potentially more ecologically valid assessment tool in comparison to externally paced protocols (Tofari et al. 2014). As such, we have adopted this approach to assess team-sport-specific running performance in the heat following cooling.

This study aimed to determine if a reduction in both core temperature and *T*_sk_ via a mixed-method pre-cooling protocol (ice slurry ingestion whilst wearing a cooling garment), improves self-paced intermittent exercise in the heat. It was hypothesised that lowering both core temperature and *T*_sk_ via mixed-method pre-cooling would enhance self-paced intermittent exercise compared to non-cooling, internal cooling or external cooling only.

## Methods

### Participants

Ten males (30.5 ± 5.8 years, 73.2 ± 14.5 kg, 176.9 ± 8.0 cm, 56.2 ± 6.6 ml/kg/min; mean ± SD), characterised as performance level 3 athletes (Pauw et al. 2013), volunteered to participate in this study. All participants trained regularly (minimum of five times per week) and were familiar with intermittent exercise (minimum exposure of intermittent exercise including, but not limited to, the following at least once per week; intermittent sports such as football and hockey, or interval training).

### Study overview

Prior to commencement of the study the University Health and Sciences Research Ethics Committee granted ethical approval (project code SH16170014-R) for all methods. All participants completed a health screen questionnaire and provided verbal and written consent to participate in the study. A repeated-measures design was used with each participant completing all interventions in a counter balanced order. Prior to the main experimental trials participants completed a graded exercise test to determine $\dot{V}{\text{O}}_{{{\text{2max}}}}$ and a separate familiarisation session of the self-paced intermittent exercise protocol. On four separate occasions participants completed an experimental trial in a temperature-controlled room (34.4 ± 1.4 °C, 36.3 ± 4.6% RH). The ambient temperature and relative humidity were similar between conditions (*p* > 0.05).

### Cooling intervention

Participants completed a 30-min pre-exercise resting period in the temperature-controlled room (34.4 ± 1.4 °C, 36.3 ± 4.6% RH), where they consumed either: (1) 7.5 g/kg of ice slurry (− 0.5 ± 0.4 °C) beverage (INT); (2) a control beverage of 7.5 g/kg of water (23.4 ± 0.2 °C) (CON); (3) wore a cooling garment whilst consuming a control beverage (23.4 ± 0.2 °C) (EXT); (4) wore a cooling garment whilst consuming the ice slurry beverage; (MIX). To ensure consistency across trials, the ice slurry was consumed in 3 equal aliquots of 2.5 g/kg/body mass every 10-min. All trial beverages contained a carbohydrate (CHO) solution (Robinson cordial, UK) to enhance palatability and were matched (0.75 g/kg of body mass) for fair comparison between trials. The cooling garment consisted of a cooling vest (Arctic Heat, Brisbane, QLD, Australia) covering the torso, worn over participants own T-shirt, and cooling towels (Frogg Toggs^®^), covering both upper and lower arms. The ice vest was stored at − 80 °C and taken out of the freezer 30-min before application. The surface temperature of the ice vests when donned by the participant was approximately 0.17 °C. The cooling towels were soaked in water and rung out 10-min before application. After 30-min, participants completed a self-paced intermittent exercise test on a non-motorised treadmill.

### Graded exercise test and familiarisation session

Participant’s height (Seca, Birmingham, UK) and body mass (Sartorius CAH3G-150IG-H’, Sartorius, Bovenden, Germany) were recorded upon arrival at the laboratory. Participants completed a 5-min self-selected warm-up prior to completing the graded exercise test for the determination of $\dot{V}{\text{O}}_{{{\text{2max}}}}$ on a motorised treadmill (h/p/cosmos mercury 4.0 h/p/cosmos sports & Medical gmbh, Nussdorf-Traunstein, Germany). Starting treadmill speed was determined from the self-selected warm-up and corresponded to a heart rate value of approximately 130 b/min. The exercise intensity increased every minute by increasing speed by 1 km/h every minute until a comfortable speed was reached and thereafter gradient was increased by 0.5% until volitional fatigue (Hamlin et al. 2012). Respiratory gases were continuously monitored throughout using an online gas analysis system (Cortex Biophysik Metalyzer, Germany) with 10-s averages being recorded. Heart rate (HR) was continuously monitored by telemetry using a HR monitor (Polar FT-1, Kempele, Finland) and rating of perceived exertion (RPE) was recorded during the last 15-s of each stage using the 6–20-point Borg Scale (Borg 1982). Blood lactate was determined from 20μL capillary blood samples (Biosen C-line, EKF Diagnostics) taken approximately 3-min post-test. The following criteria; a plateau in $\dot{V}{\text{O}}_{{{\text{2}}}}$, HR ≥ 85% age predicted heart rate max, RER > 1.15, RPE ≥ 19, voluntary exhaustion, post blood lactate ≥ 8.0 mmol/L was used to determine $\dot{V}{\text{O}}_{{{\text{2max}}}}$. This was in accordance with the criteria recommended by BASES (1997). If all criteria were not met then a V̇O_2peak_ value was recorded as the mean value from the final 30 s.

With at least 24-h separating the $\dot{V}{\text{O}}_{{{\text{2max}}}}$ test, to ensure adequate test–retest reliability (Tofari et al. 2014), participants returned to the laboratory ( ~ 19 °C, 45% RH) to familiarise themselves with the intermittent protocol on the non-motorised Treadmill (Woodway Curve 3.0TM; Woodway, Inc., Waukesha, Wisconsin, USA).

A 46-min modified protocol derived from the 30-min self-paced intermitted protocol, validated by Tofari et al. 2014 was employed, which has a coefficient of variation (with 90% confidence intervals) from a test–retest (*n* = 3) of 2.9% (3.8–4.1) for total distance covered (km), 4.6% (3.3–8.1) for peak speed (km/h) and 3.8% (0.96–1.1) for sprint distance (km).

### Exercise protocol

The protocol included a 2-min warm-up at a self-selected speed followed by a 46-min intermittent protocol to replicate the first half of a football game. This was based on a previously published protocol (Gerrett et al. 2017). The protocol consisted of three 15.5-min periods separated by a 71 s recovery period and included the following; 10 × 10 s sprints, 3 × 51 s run, 6 × 50 s jog, 6 × 30 s walk, and 2 × 30 s rest period. The high-intensity bouts (run and sprint) were always followed by a low-intensity bout (rest, walk or jog). Participants were informed that the performance indicators were the total distance covered and their average speed during each bout and their maximal speeds during ‘sprint’ and ‘run’ commands. Participants were instructed that ‘sprints’ should be completed at 100% effort, ‘runs’ at 75% effort, ‘jogs’ at 45% effort and ‘walks’ at 10% effort. A screen was placed at eye level and displayed the exercise instruction (e.g., WALK, JOG, RUN, and SPRINT), the total exercise time and a time indicator of when the next exercise instruction would appear. Information regarding speed and distance covered were concealed from view.

### Experimental protocol

Participants were instructed to swallow a telemetric pill (Cortemp; HQ Inc., Palmetto, FL, USA) 7–8 h prior to each test session. On arrival at the laboratory a urine sample was collected prior to measurement of semi-nude body weight. Eight wireless *T*_sk_ sensors were attached and a HR monitor worn, after which, a resting fingertip blood lactate sample was taken along with a whole-body thermal sensation (modified ASHRAE scale 2005). Participants then sat for 30-min in temperature-controlled room (34.4 ± 1.4 °C, 36.3 ± 4.6% RH) and completed INT, CON, EXT or MIX, as described above. At 5-min intervals during the pre-exercise period *T*_gi_, HR, and thermal sensation were recorded.

Within 5-min of consuming the final beverage and/or removing cooling garments, participants began the intermittent exercise protocol (as outlined above) in the same temperature-controlled room with a fan set at 1.3 m/s placed 75 cm in front of the participant directed towards the torso. At 5-min intervals *T*_gi_, HR, RPE, and thermal sensation were recorded. During the two 71 s recovery period (mid 1 and mid 2) and at the end of the test (post) a blood lactate sample was taken from the fingertip. At the end of the exercise protocol, participants towel-dried themselves and were weighed semi-nude, prior to the collection of a post urine sample. All trials were completed at the same time of day ( ± 1 h), with at least 5 days separating trials. Participants were asked to replicate their diet prior to each visit and refrain from caffeine 12-hr and alcohol 24-hr preceding the trials.

## Measurements

### Exercise performance data

Running speed and distance covered were recorded continuously (sampling at 100 Hz) during the 46-min intermittent protocol on a non-motorised treadmill. The average speed and total distance covered during each exercise profile (WALK, JOG, RUN, and SPRINT) were calculated. The peak speed achieved during the high-intensity activity profiles (RUN and SPRINT) were identified. The mean velocity in each stage (WALK, JOG, RUN, and SPRINT) was expressed as a percentage of the individuals peak velocity achieved during that trial. The aforementioned data were separated into the first, second, and third (15.5-min) periods. The total distance covered for the entire protocol was also calculated for each condition.

### Core, skin, and body temperature

The telemetric pill was used to measure gastrointestinal temperature (*T*_gi_), which was used as an indicator of core temperature. This was measured every 5 min during the pre-exercise and exercise period. A safety stop criteria was set at 39.5 °C but this was not exceeded by any participants in any conditions. Based on the recommendations by Hunt et al. (2017) all telemetric pills were checked against a reference mercury thermometer in a water bath and where appropriate corrections were applied to reduce any systematic bias. *T*_sk_ was measured at eight sites (forehead, chest, upper back, upper arm, forearm, hand, thigh, and calf) using iButtons^®^ (Maxim Integrated Products, Inc., Sunnyvale, California, USA). Occasionally some data (1 or 2 locations) were missing due to equipment errors so an unweighted mean *T*_sk_ was calculated. *T*_sk_ was continuously recorded at a sample rate of 1 per second with data averaged over a 5-min period. Mean body temperature (*T*_b_) was calculated as 0.79 × * T*_gi_  + 0.21 × mean *T*_sk_ (Colin et al. 1971).

### Thermal sensation, RPE, heart rate, and blood lactate

HR was recorded using a wireless HR monitor (Polar FT-1), sampling every second and averaged every 5-min during rest and exercise. Thermal sensation was rated using a scale ranging from + 10 (extremely hot) to − 10 (extremely cold) with 0 indicating thermal neutrality (Ashrae 2005) and was recorded at 5-min intervals during rest and exercise. Participants were instructed to report the number (corresponding to the thermal sensation description) that best represented their whole-body thermal status at that moment in time and were not permitted to recall their previous sensation. RPE was recorded using the 6–20-point Borg Scale (Borg 1982) at 5-min intervals during exercise only. Participants were instructed to report the number (corresponding to the exertion description) that best represented how hard they felt they worked for the preceding 5-min period. TS and RPE were collected by the same researcher and accompanied by standardised instructions and the memory-anchoring procedure (Haile et al. 2014). Capillary blood samples (20 µL) were taken from the fingertip and analysed for blood-lactate concentration.

### Urine osmolality and gross sweat loss

Urine samples were assessed for urine osmolality using a portable osmometer (Vitech Scientific, West Sussex). Changes in semi-nude body mass were used to estimate gross sweat loss (g) adjusted for fluid intake.

### Statistical analysis

Before fitting statistical models, a data exploration was undertaken following a protocol to visualise and examine the data for outliers in both response and explanatory variables, homogeneity in the response variables, collinearity between explanatory variables, and the nature of the relationships between response variables and explanatory variables (see Zuur et al. 2009). No influential outliers were found and the data met the assumptions required for the chosen statistical models. Data exploration was performed using R statistical software (R Core Team 2017). Performance variables (distance covered, average speed and maximum speed, and percentage of peak speed) were investigated using linear mixed effects models. Condition (INT, CON, EXT or MIX), activity (WALK, JOG, RUN, SPRINT) and period (first, second, third) were treated as fixed effects. The model included an interaction term that allowed the quantification of the interaction between conditions, activities and periods, and a random term was used to estimate variation between participants whilst also controlling for the treatment and activity effects.

Changes in the physiological responses were used for the main part of the analysis and the average absolute data are presented in supplementary files. Generalised Additive Mixed Models (GAMMs) were fitted to the data for the various physiological responses to treatment and activity due to non-linear patterns in the temperature change-response variable identified at the data exploration stage. GAMMs incorporate non-parametric smooth terms with mixed effects (Wood 2017). Condition and time were treated as fixed effects, including a time × condition interaction term. A random term was used to estimate variation between participants whilst also controlling for the treatment and activity effects. The model was fitted to the data to test the effects of different pre-cooling treatments on changes in the physiological responses of the participants. Smooths were fitted as interaction terms with different treatments to investigate if patterns in physiological responses were significantly different between treatments. 95% CIs were used as a way of confirming significance during the trials whereby if the intervals do not overlap at all then a real effect has occurred (O’Brien and Yi 2016). All models were fitted using the gamm4 package (Wood and Scheipl 2017) in R. The assumptions for all models were checked by extracting residuals and plotting these with fitted values to check for non-linear patterns and homogeneity of variance. Residuals were also plotted against covariates used within the model as a further check for homogeneity and any potential patterns. Residual patterns were absent and homogeneity of variance satisfied.

Comparison between pre-cooling protocols was standarised using Cohens d effect sizes (ES) with the following descriptive criteria; an ES of < 0.2 is classified as ‘trivial’, 0.2–0.4 as ‘small’, 0.5–07 as ‘moderate’ and > 0.8 as a ‘large’ effect.

## Results

**Fig. 1:**
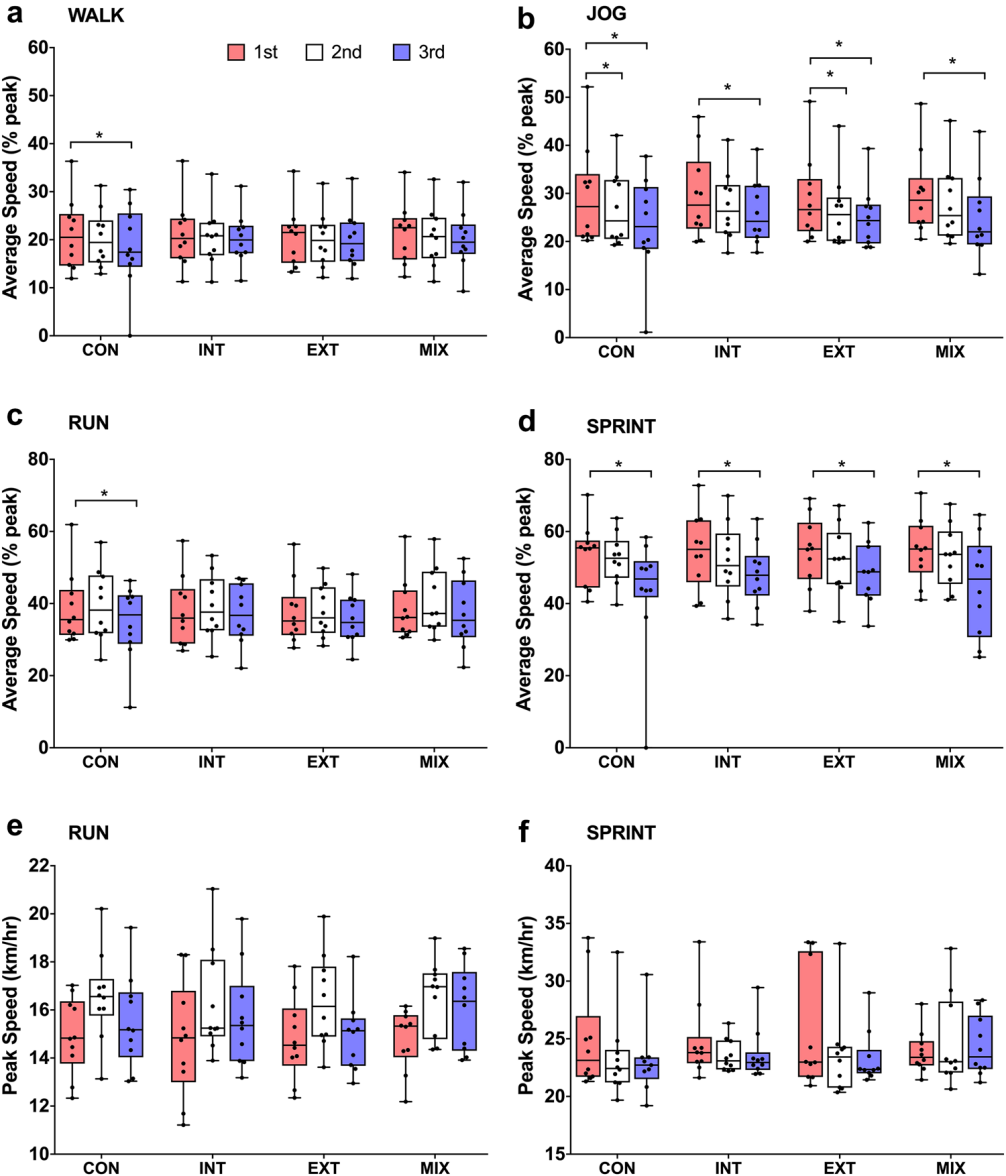
Box plots comparing the percentage of peak speed (**a**–**d**) for the WALK, JOG, RUN, and SPRINT during the first period, second period and third period in the four conditions (*CON* control, *INT* internal cooling, *EXT* external cooling, *MIX* internal, and external cooling). Peak speeds during the RUN and SPRINT are also shown (**e**, **f**). Boxes indicate the 25th and 75th percentiles. Whiskers indicate minimum and maximum values with the middle line representing the median. Individual data points are indicated by black dots. For peak speeds there were no significant effects of condition, time or an interaction effect (*p* > 0.05). There was no interaction effect (*p* < 0.05). The percentage of peak speed declined for some conditions from the first to second period (^#^*p* < 0.05) and first to third period (*p < 0.05)

**Fig. 2:**
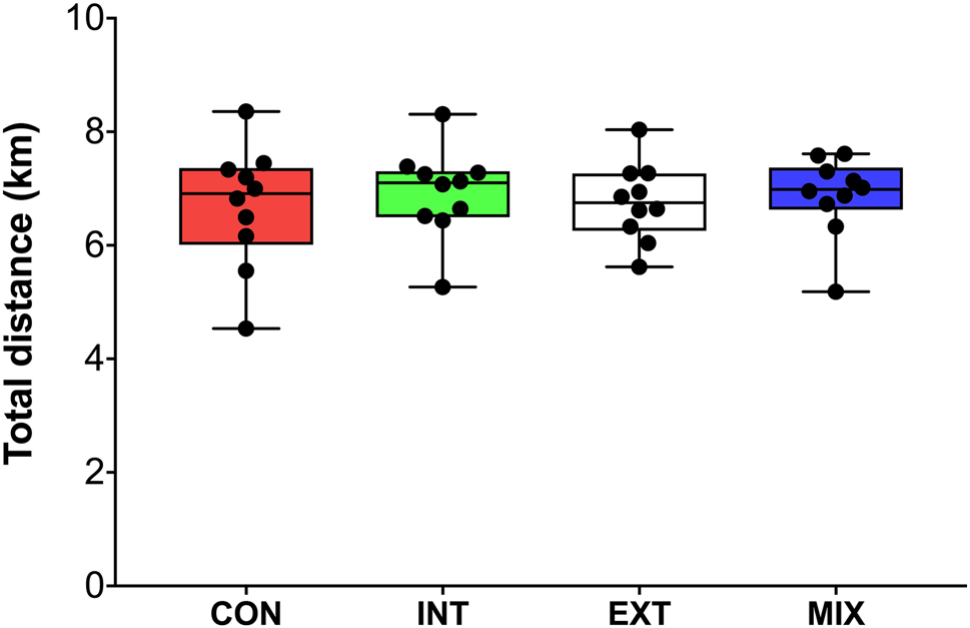
Box plot illustrating the total distance covered in the four conditions (*CON* control, *INT* internal cooling, *EXT* external cooling, *MIX* internal, and external cooling). Boxes indicate the 25th and 75th percentiles. Whiskers indicate minimum and maximum values with the middle line representing the median. Individual data points are indicated by coloured dots

**Fig. 3:**
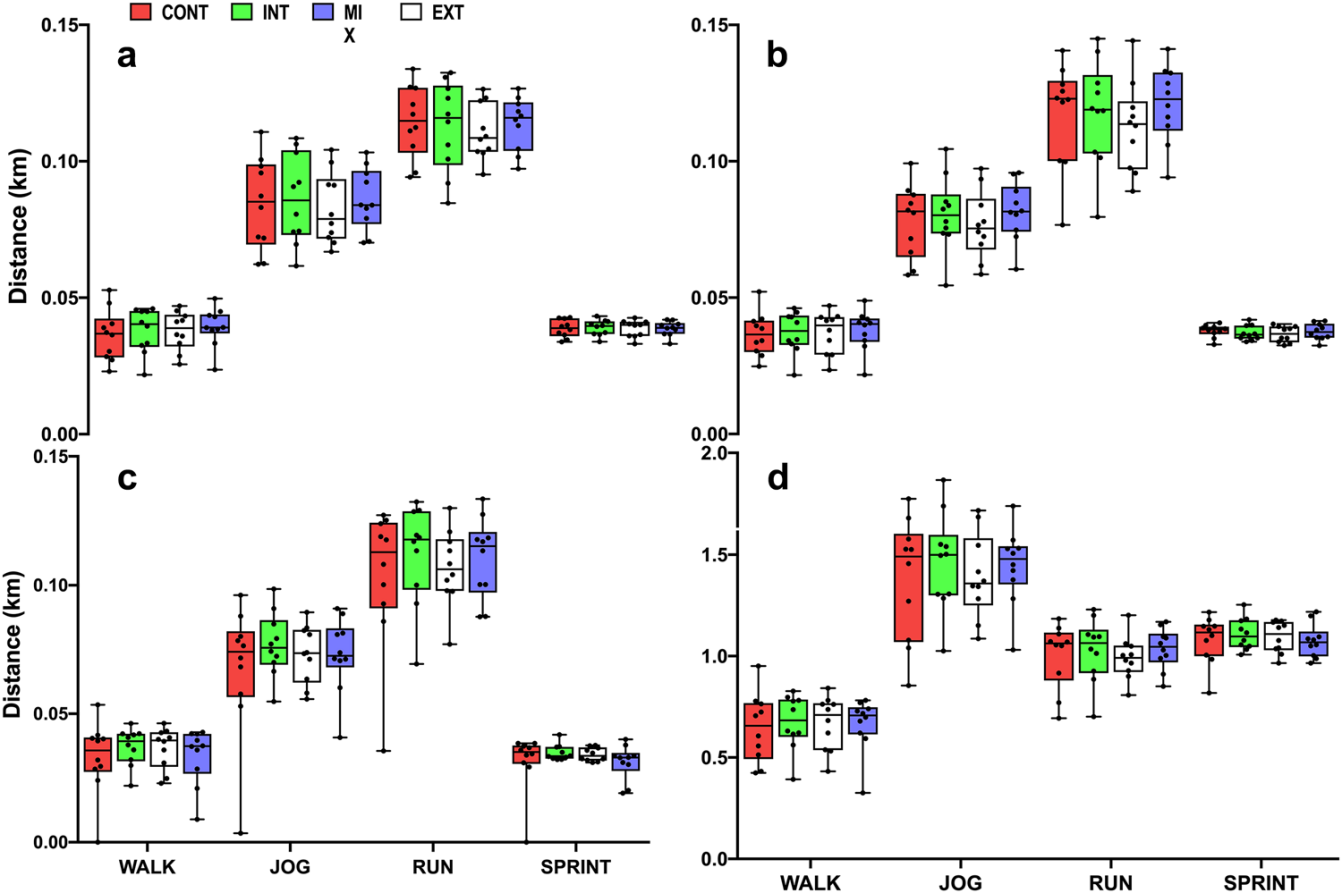
Box plots comparing the accumulated distances during the **a** first period, **b** second period, **c** third period, **d** total distance in the first, second and third periods for each activity profile (walk, jog, and run sprint) covered in the four conditions (*CON* control, *INT* internal cooling, *EXT *external cooling, *MIX* internal, and external cooling). Boxes indicate the 25th and 75th percentiles. Whiskers indicate minimum and maximum values with the middle line representing the median. Individual data points are indicated are indicated by black dots. The distance covered during SPRINT were greater during the first and second period compared to the third (*p* < 0.05). There was no interaction effect (*p* < 0.05)

The mixed model showed that there was no effect of condition on average speed (Fig. 1a–d) nor peak speed (Fig. 1e, f) (*p* > 0.05). Trivial to small effect sizes were observed between conditions (ES = 0.01–0.23). Total distances covered (Fig. 2) were similar between conditions (CON: 6.69 ± 1.08 km, INT: 6.96 ± 0.81 km, EXT: 6.76 ± 0.65 km, MIX 6.87 ± 0.70 km) (*p* > 0.05) and only small effect sizes observed between CON vs. INT (ES = 0.28) CON vs. MIX (ES = 0.20) and INT vs. EXT (ES = 0.26). There were no significant differences in the distance covered between conditions for each activity profile (Fig. 3a–d, *p* > 0.05) with trivial-to-small effects observed (ES = 0.1–0.42).

There was a significant decline in all performance variables (peak speeds, percentage of peak speeds, and distance covered) from period 1 to period 3 (*p* < 0.05); however, there was no interaction with condition or activity. The significant decline from the first period to the third period in peak speed achieved during the RUN and SPRINT (*p* < 0.05) had only small to moderate effect sizes (ES = 0.20–0.59). For the percentage of peak speed (Fig. 1a–d) for all conditions, effect sizes between the first and third period for the WALK and RUN were small to trivial (ES = 0.13–0.45 and 0.10–0.40, respectively) and small-to-moderate for JOG (ES = 0.43–0.60). Whilst moderate-to-large effect sizes were observed for SPRINT activity profile between the third period compared to the first (ES = 0.67–0.87). The decline in distances covered between the first and third period was small for the WALK (ES = 0.3) and RUN (ES = 0.29), moderate for JOG (ES = 0.79) and large for SPRINT (ES = 1.11).

**Fig. 4:**
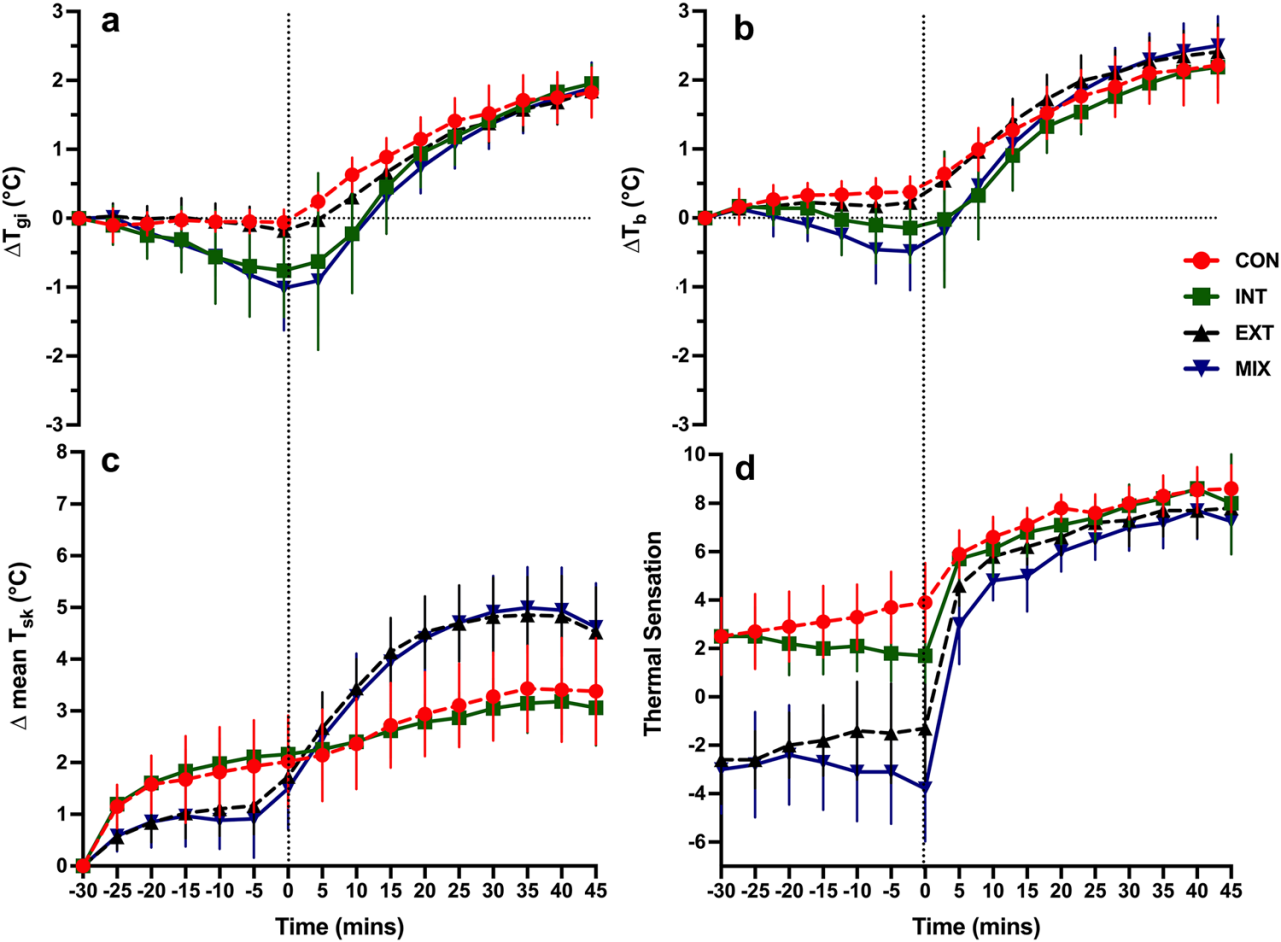
Δ mean *T*_gi_, *T*_b_, and mean *T*_sk_ (**a–c**) and mean TS (**d**) during the *CON* (control), *INT* (internal cooling), *EXT* (external cooling) and *MIX* (mixture of internal and external cooling). Vertical lines indicate 95% confidence intervals

The models for change in physiological responses (*T*_gi_, *T*_b_, and mean *T*_sk_) showed significant effects of time (Fig. 4) (*p* < 0.001). On inspection of the 95% confidence intervals, compared to CON, changes in *T*_gi_ for MIX and INT was significantly different at the end of pre-cooling and at start of exercise. EXT and MIX was also significantly different from one another at the end of pre-cooling and at start of exercise (− 5 to 5) as evidenced by a lack of CI overlap (Fig. 4a). Similar changes in *T*_gi_ across all conditions were evident after 10 min. There were moderate to large effect sizes (ES > 0.5) for differences between CON vs. INT (from − 10 to + 10 min), CON vs MIX (from -10 to 15 min), EXT vs, MIX (from − 15 to 15 min) and INT vs EXT from -20 to 10 min into exercise (ES = 0.5–0.77). Small to trivial effects were observed throughout the protocol for INT vs. MIX (ES < 0.30) and CON vs. EXT (ES < 0.36) except the final 5 min of exercise (ES > 0.57). A greater rise in *T*_gi_ and the rate of rise ( °C /min) were observed for INT (∆*T*_gi_: 2.7 ± 1.1 °C and 0.057 ± 0.04 °C /min) and MIX (∆*T*_gi_: 3.0 ± 1.1 °C and 0.062 ± 0.02 °C /min) compared to EXT (∆*T*_gi_: 2.0 ± 0.5 °C and 0.041 ± 0.008 °C /min) and CON (∆*T*_gi_: 1.8 ± 0.4 °C and 0.042 ± 0.01 °C/min) (*p* < 0.05) as *T*_gi_ were significantly lower at the start of the exercise protocol but *T*_gi_ were similar between conditions at the end of exercise.

Differences in T_b_ change (Fig. 4b) were evident between CON and MIX, especially between − 20- and 5-min. T_b_ for all other conditions were similar between pre-cooling and exercise. Moderate-to-large effect sizes (ES > 0.5) were observed between CON vs. INT (0–10 min), CON vs. EXT (− 30 to 10 min), CON vs. MIX (− 20 to 15 min), INT vs. EXT (− 30 to − 15 and from 35–45 min), INT vs. MIX (− 30 to 5 min), and EXT vs. MIX (from − 10 to 10 min and from 40 to 45 min).

Mean *T*_sk_ increased for all conditions and were higher than baseline throughout the entire protocol (*p* < 0.05). In comparison to baseline, large effects were observed from − 5 to 0 and then from − 15 until the end of the experiment (ES > 0.54). Differences in mean *T*_sk_ change were evident between CON and EXT and CON and MIX and found between 20 and 25 min and between 25 and 30 min, respectively (*p* < 0.05). Whereas CON and INT followed a similar pattern of increase. Differences in mean *T*_sk_ change were also evident between INT and EXT, and INT and MIX, these differences were found between 15 and 40 min, and between 20 and 40 min, respectively (*p* < 0.05). Moderate-to-large effect sizes (ES > 0.5) were observed between CON vs. INT (− 25 to 10 min), CON vs. EXT (− 30 to 20 min), CON vs. MIX (− 30 to 15 min), INT vs. EXT (throughout the entire protocol), INT vs. MIX (− 30 to 15 min), and EXT vs. MIX (from − 30 to − 15 min and from 10 to 45 min.

The model for thermal sensation showed significant changes through time for all conditions (*p* < 0.05). As soon as exercise started thermal sensation increased quickly for all conditions but the lower the thermal sensation score at the end of pre-cooling, the greater the increase in thermal sensation upon the initiation of exercise (∆thermal sensation from 0 to 5 min): CON: 2 ± 1.7, INT: 4 ± 1.6, EXT: 5.9 ± 2.3, MIX: 6.8 ± 2.3). From 0 to 5 min, large effect sizes were noted for all conditions (ES > 0.81) and these ES were larger for the conditions which have the lowest TS at 0 min (ES: CON = 0.81, INT = 1.26, EX = 1.86, MIX = 1.97). Significant differences and moderate effect sizes (ES > 0.5) in thermal sensation were evident during pre-cooling (− 30 to 0 min) for both CON and EXT, and CON and MIX. Significant differences in TS were also evident during pre-cooling for both INT and EXT (− 30 to − 5 min), and INT and MIX (− 30 to 5 min). Whereas TS for CON and INT, and EXT and MIX were similar throughout the pre-cooling period (*p* > 0.05, ES < 0.2). During exercise, after 5 min, all conditions were similar and followed a similar pattern of increase.

HR, blood lactate, and RPE data are illustrated in Table 1. HR and RPE were similar between all conditions and there were no effects of condition but an effect of time as all data during exercise were higher than baseline. Trivial-to-small effects were noted for HR and RPE between conditions throughout the entire protocol (ES < 0.4). There were moderate-to-large effects between exercise data and pre-exercise measures for all conditions (ES > 5.0). Blood lactate was significantly lower pre-and there was weak evidence of lower lactate levels for the EXT treatment in the Mid1 and 2. Post blood lactate levels for CON were significantly higher than INT and marginally higher than the EXT and MIX. Trivial-to-small effects were noted between conditions at pre, mid 1, mid 2 and post (ES < 0.28) and large effects were observed between exercise data and baseline for all conditions (ES > 1.9).

**Table 1:** Mean ( ± SD) heart rate, blood lactate and RPE measured at baseline (BL) and at 5-min intervals during exercise

Time (min)		0	5	10	15	20	25	30	35	40	45
Heart rate (bpm)											
CON		63 ± 15	158 ± 16	162 ± 18	157 ± 22	170 ± 14	168 ± 15	170 ± 14	170 ± 18	172 ± 19	173 ± 17
INT		66 ± 11	157 ± 21	164 ± 14	144 ± 19	158 ± 19	166 ± 18	167 ± 14	169 ± 16	171 ± 15	170 ± 14
EXT		61 ± 12	153 ± 20	160 ± 20	154 ± 24	164 ± 23	168 ± 18	164 ± 18	167 ± 21	171 ± 16	171 ± 14
MIX		61 ± 10	160 ± 12	162 ± 13	161 ± 18	172 ± 13	170 ± 15	172 ± 12	171 ± 11	176 ± 13	170 ± 25
RPE											
CON		–	13 ± 2	15 ± 2	15 ± 2	16 ± 2	17 ± 1	17 ± 1	17 ± 2	18 ± 2	18 ± 2
INT		–	14 ± 1	15 ± 1	15 ± 2	16 ± 1	16 ± 1	17 ± 2	17 ± 2	18 ± 2	18 ± 2
EXT		–	13 ± 1	14 ± 1	15 ± 1	16 ± 2	16 ± 2	16 ± 2	17 ± 2	18 ± 2	18 ± 2
MIX		–	13 ± 1	14 ± 1	15 ± 1	16 ± 1	16 ± 2	16 ± 1	17 ± 2	17 ± 2	17 ± 2
Blood lactate (mmol/L)		BL			Mid1			Mid2			Post
CON		1.5 ± 0.6	–	–	6.4 ± 3.1	–	–	6.5 ± 2.9	–	–	6.2 ± 2.4
INT		1.4 ± 0.4	–	–	6.6 ± 3.1	–	–	6.6 ± 3.1	–	–	5.9 ± 1.8
EXT		1.5 ± 0.4	–	–	5.6 ± 3.1	–	–	6.1 ± 3.3	–	–	6.1 ± 2.6
MIX		1.4 ± 0.4	–	–	6.8 ± 3.1	–	–	6.1 ± 2.6	–	–	6.0 ± 2.0

Data collected in all 4 conditions are presented (CON: control, INT: internal cooling, EXT: external cooling and MIX: internal and external cooling). Blood lactate was measured at BL and at 3 interspersed time points during the exercise protocol (Mid 1, Mid 2, and Post exercise). Heart rate, blood lactate and RPE all increased above baseline during exercise (*p* < 0.05) but there were no differences between conditions nor an interaction effect (*p* > 0.05)

Gross sweat loss (GSL) were similar between conditions: CON 719.9 ± 211.9 g/min, INT 738.6 ± 181.9 g/min, EXT 696.0 ± 162.9 g/min, MIX 691.1 ± 182.5 g/min (*p* < 0.05, ES < 0.20).

## Discussion

This study aimed to determine if lowering both *T*_gi_ and *T*_sk_ using a combined pre-cooling protocol (ice slurry ingestion whilst wearing a cooling garment) improved self-paced intermittent exercise in the heat. We hypothesised that a mixed pre-cooling protocol would lower both *T*_gi_ and *T*_sk_ more than internal or external cooling alone. The benefit of which would increase the heat storage capacity and enhance self-paced intermittent exercise performance. MIX was effective in reducing *T*_gi_ and *T*_sk,_ and thus T_b_ (prior to exercise) in comparison to all other conditions. In addition, thermal sensation was significantly lower during MIX compared to the CON and INT conditions, but was similar to EXT. Despite these aforementioned thermophysiological responses prior to exercise, there were no performance benefits during low-, moderate- or high-intensity efforts at any time point during the 46-minute protocol (*p* < 0.05).

### Core and skin temperature

As expected *T*_gi_ did not change during pre-cooling for CON or EXT but by the end of pre-cooling *T*_gi_ had dropped by − 0.79 ± 0.9 °C and − 0.96 ± 0.8 °C for INT and MIX, respectively. The additive effect of combining ice-slurry ingestion with an external cooling garment resulted in a 0.17 °C reduction in *T*_gi_ in comparison to internal cooling alone. This suggests that the addition of an external cooling garment alongside internal cooling can provide a small but meaningful change in *T*_gi_. However, after 15-min of self-paced intermittent exercise *T*_gi_ was similar between conditions. Unlike endurance performance studies where core temperature is usually higher at the end of exercise in the pre-cooled compared to controlled condition (Siegel et al. 2010, 2012), *T*_gi_ at the end of the intermittent exercise protocol in our study was similar between all conditions. This seems to be a common trait amongst studies that successfully lowered core temperature prior to intermittent exercise (Aldous et al. 2018; Duffield and Marino 2007; Gerrett et al. 2017).

Whilst we were able to successfully lower mean *T*_sk_ using the external cooling techniques (MIX and EXT), this only lasted somewhere between 10 and 20-min during exercise. Mean *T*_sk_ was similar after only 10-min of exercise during MIX compared to CON but had the longest effect (20 min) for EXT compared to INT. From our results and previous literature, it seems that with the start of intermittent-based exercise the benefits of external cooling on mean *T*_sk_ are short lived with convergence to a similar mean *T*_sk_ occurring within the first 10 min (Duffield and Marino 2007; Price et al. 2009; Skein et al. 2012). A common trend that appears across external cooling studies is that the rate of rise in *T*_sk_ is typically greater for an external cooling technique compared to a control or internal cooling technique (Castle et al. 2006; Duffield and Marino 2007; Minett et al. 2011; Stevens et al. 2017). This greater rate of change in *T*_sk_ will increase the firing rate of warm thermoreceptors; resulting in stronger thermal perceptual responses (de Dear et al. 1993; Zhang et al. 2004). This is further supported from our thermal sensation responses where we typically observed that the lower the thermal sensation score at the end of pre-cooling, the greater the increase (and larger ES) in thermal sensation upon the initiation of exercise. This increase in perception of warmth may bestow no performance benefit via behavioural thermoregulation (Schlader et al. 2011). Internal cooling has been reported to delay the onset time of thermo-effector responses (i.e. sweating/vasodilation starts later into exercise) leading to a more rapid accumulation of metabolic heat during the early phase of exercise despite a lower absolute core temperature (Jay and Morris 2018). Whilst no sudomotor or vasomotor responses were measured in the present study and we also observed no differences in GSL between conditions, we did observe a greater rise in *T*_gi_ for the two conditions that employed an internal cooling protocol (INT and MIX) compared to external only and control conditions. The external cooling techniques also resulted in a greater rate of change in *T*_sk_ for EXT and MIX conditions. Maintaining a lower *T*_sk_ is important to provide a more efficient heat transfer gradient from the core.

Aldous et al. (2018) maintained the effects of mixed-method pre-cooling (7.5 g/kg of ice slurry at − 1 °C and ice vest and towel to torso and arms and ice packs around the upper legs) on core temperature, *T*_sk_ and thermal sensation throughout the first half (45-min) of an intermittent protocol compared to a control condition. Whilst in our study, these thermophysiological responses were similar to the control within 10–15 min of exercise. We conducted our pre-cooling within the temperature-controlled room (34.4 ± 1.4 °C, 36.3 ± 4.6% RH), whereas Aldous et al. (2018) conducted all cooling manoeuvres in a temperate condition (18 ± 0.9 °C, 50.3 ± 4.7% RH) before moving into hot conditions for exercise (30.7 ± 0.3 °C, 50.9 ± 4.2% RH), which may have offered a prolonged cooling advantage. This suggests pre-cooling in an already cool room may be more effective than cooling in the same hot environment. The influence of the environmental conditions where pre-cooling techniques are administered requires clarification.

## Performance

We previously reported no performance benefit of internal cooling on a similar (but shorter) self-paced intermittent protocol compared to a controlled beverage (Gerrett et al. 2017). We speculated that the minimal effect of internal cooling on mean *T*_sk_ may have accounted for no performance improvement due to the reported role of *T*_sk_ on behavioural regulation (Faulkner et al. 2015; Sawka et al. 2011; Schlader et al. 2011). As a result, in the present study we investigated whether the addition of external cooling with internal cooling would benefit self-paced intermittent performance, especially if the combined techniques lowered both core and *T*_sk_. Whilst we observed an increased heat storage capacity and lower thermal perception by combining internal and external cooling it offered no benefit, as performance variables were similar between conditions. More surprisingly, the performance variables were all similar between conditions during the first 15 min of the protocol where significant and/or large effect sizes were observed in *T*_gi_ between both the INT and MIX and EXT and CON conditions.

This is not the first study to show no improvements in intermittent exercise performance following reductions in only core temperature (Gerrett et al. 2017; Zimmermann and Landers 2015) and a meta-analysis reported a small effect size of lowering core temperature prior to intermittent exercise (Hohenauer et al. 2018). The evidence of lowering both core temperature and *T*_sk_ has been more equivocal with some evidence reporting a performance benefit for low intensity but not moderate-maximal efforts (Duffield and Marino 2007; Minett et al. 2011) and others vice-versa (Aldous et al. 2018). Aldous et al. (2018) found moderate improvements in high speed (very likely, 0.68) and a variable run (very likely, 0.81) distances covered during the first half (45 min) of a simulated soccer match. They attributed this to reductions in core temperature, *T*_sk_ and thermal sensation, which were maintained throughout the 45-min intermittent protocol. Maintaining lower thermophysiological responses during exercise may be important for attenuating the loss of performance in hot conditions. This may explain the discrepancy between the two studies.

We observed lower percentage of peak speeds in the final (third) 15.5-min period compared to the first 15.5-min period for submaximal running (WALK, JOG and RUN) during CON only. This suggests that there may be very subtle effects upon performance that were apparent in moderate and high intensity exercise, but not sprinting. INT and MIX cooling strategies further enhanced the attenuation in peak speed between the first and third period whilst jogging. Maximal intensity exercise (SPRINT) had a significantly lower percentage of peak speed and large ES ( > 0.67) during the third period in comparison to the first period for all conditions. This is reflective of a down regulation of exercise intensity perhaps due to incremental fatigue during intermittent exercise.

Brade et al. (2013) also found no significant improvement in intermittent exercise performance following a combined internal and external precooling protocol. The effects of mixed-methods pre-cooling on intermittent-sprint performance may only become apparent when the thermal stress is sufficient to induce heat strain (Duffield and Marino 2007). The RH (36.3 ± 4.6% RH) in the current study and Brade et al. (2013) (37–38% RH) may not have been of sufficient magnitude for pre-cooling to have any positive effect. Chmura et al. (2017) observed the activity profiles of 2014 FIFA World Cup players and found that when RH was above 60% the number of sprints were significantly reduced, regardless of air temperature (below 22 °C or above 28 °C). It is speculated that when the environmental conditions suppress heat dissipation (i.e. > 60%RH) the benefits of pre-cooling may be more apparent. The environmental conditions could strongly influence core temperature and recently it has been suggested that intermittent sprint performance is impaired when core temperature exceeds 39 °C and as such one should aim to attenuate the core temperature rise (Girard et al. 2015). Our data and previous studies indicate that the environmental conditions of the activity and the conditions in which pre-cooling is employed, can impact upon whether pre-cooling results in detectable benefits in intermittent sprint performance (Aldous et al. 2018; Brade et al. 2013; Duffield and Marino 2007; Gerrett et al. 2017). As such, further research may be necessary to confirm the specific conditions in which pre-cooling may or may not be effective.

### Sensory feedback

The role of *T*_sk_ on behavioural regulation has been documented in endurance-based exercise activities (Schlader et al. 2011) but not intermittent exercise. However, we speculated that by lowering both *T*_gi_ and *T*_sk_ a colder thermal sensation would be experienced which would benefit behavioural thermoregulation and allow for improved performance compared to a control condition. However, the benefits of lowering thermal sensation with our external cooling techniques were short-lived, converging within 10 min and having the greatest rate of change for MIX compared to CON and INT. Although MIX lowered both *T*_gi_ and mean *T*_sk_, the thermal sensations were similar to EXT which lowered mean *T*_sk_ only. External cooling is therefore required to alter thermal sensation but combining it with an internal cooling technique did not seem to enhance thermal sensation in our study. Favourable thermal perceptions may need to be strong enough to attenuate performance decrements in the heat, as such, maintaining a lowered core temperature, mean *T*_sk_ and thermal sensation may be required for intermittent exercise performance in the heat. This warrants investigation.

## Limitations

Whilst an extended warm-up is typical practice for most athletes, to determine the respective effects of the pre-exercise cooling interventions the warm-up in the current study was restricted to 2 min. This was to minimize the time between the pre-cooling and commencement of the protocol and maximize the physiological and perceptual effects of the cooling and elucidate any controlling mechanisms. However, it should be highlighted that this is not an externally valid approach for team sport athletes but others have investigated cooling strategies integrated into sport-specific warm ups (Taylor et al. 2019). It should be noted, despite speed and distance covered being similar across conditions altered thermal perception may affect decision-making and skill execution, which is considered a key component of success during intermittent team sports such as football. The influence of precooling on decision-making and skill execution warrants further investigation.

## Conclusions

This study demonstrated a practical mixed-method pre-cooling technique does not improve self-paced intermittent exercise in hot dry conditions. Despite lowering *T*_gi,_ mean *T*_sk_ and thermal sensation prior to exercise, the distance covered during submaximal and maximal exercise bouts were similar to conditions that offered no cooling or lowered *T*_gi_, or mean *T*_sk_ only. The benefits of pre-cooling on thermophysiological responses waned after 10–20 min. Our data and evidence from previous studies indicate that pre-cooling techniques should be employed that can attenuate the increase in thermophysiological and thermal-perceptual responses during exercise to enhance performance. Further research is required to investigate if pre-cooling strategies can enhance self-paced intermittent exercise in hot and humid conditions.

### Author contribution statement

GT and NG conceived and designed the research. GT, MD, CH and BD carried out the experiment. NG analysed the data. GT, TC, BD and NG wrote the manuscript. NG supervised the project. All authors read and approved the manuscript.

## Electronic supplementary material

Below is the link to the electronic supplementary material.

Supplementary material 1 (DOCX 20 kb)

## Abbreviations

ANOVA: Analysis of variance
CON: Control; Control beverage of 7.5 g/kg of water
ES: Effect size
EXT: External cooling; Wore a cooling garment whilst consuming a control beverage
HR: Heart rate
INT: Internal cooling; 7.5 g/kg of ice slurry beverage
MIX: Mix cooling; Wore a cooling garment whilst consuming ice slurry beverage
RH: Relative humidity
*T*_gi_: Gastrointestinal temperature
*T*_b_: Body temperature
*T*_sk_: Skin temperature
$\dot{V}{\text{O}}_{{{\text{2max}}}}$: Maximum oxygen uptake
