# Supplementary material for: Independent or simultaneous lowering of core and skin temperature has no impact on self-paced intermittent running performance in hot conditions
Source: Eur J Appl Physiol. 2019 Jun 20;119(8):1841–53. doi: 10.1007/s00421-019-04173-y (PMC6647662; doi:10.1007/s00421-019-04173-y)
Supplement: Supplementary file 1 — Supplementary material 1 (DOCX 20 kb) [file 421_2019_4173_MOESM1_ESM.docx]

| Time (min) |  | - 30 | - 25 | - 20 | - 15 | - 10 | - 5 | 0 | 5 | 10 | 15 | 20 | 25 | 30 | 35 | 40 | 45 |
| --- | --- | --- | --- | --- | --- | --- | --- | --- | --- | --- | --- | --- | --- | --- | --- | --- | --- |
| T_gi_ | CON | 37.12 ± 0.62 | 37.02 ± 0.64 | 37.02 ± 0.54 | 37.07 ± 0.55 | 37.04 ± 0.52 | 37.03 ± 0.49 | 37.02 ± 0.48 | 37.29 ± 0.49 | 37.62 ± 0.45 | 37.91 ± 0.54 | 38.16 ± 0.58 | 38.38 ± 0.58 | 38.54 ± 0.53 | 38.72 ± 0.44 | 38.78 ± 0.43 | 38.87 ± 0.43 |
|  | INT | 37.13 ± 0.38 | 37.05 ± 0.38 | 36.79 ± 0.62 | 36.7 1 ± 0.73 | 36.43 ± 1.11 | 36.24 ± 1.33 | 36.18 ± 1.30 | 36.22 ± 1.98 | 36.91 ± 1.08 | 37.53 ± 0.88 | 38.09± 0.55 | 38.18± 0.65 | 38.55 ± 0.37 | 38.68 ± 0.36 | 38.88 ± 0.41 | 39.02 ± 0.36 |
|  | EXT | 37.08 ± 0.52 | 37.11 ± 0.44 | 37.07 ± 0.46 | 37.08 ± 0.33 | 37.03 ± 0.34 | 36.98 ± 0.36 | 36.90 ± 0.47 | 37.08 ± 0.31 | 37.40 ± 0.44 | 37.77 ± 0.39 | 38.04 ± 0.30 | 38.29 ± 0.28 | 38.4 2± 0.32 | 38.60 ± 0.32 | 38.70 ± 0.32 | 38.86 ± 0.31 |
|  | MIX | 37.08 ± 0.55 | 37.08 ± 0.52 | 36.90 ± 0.70 | 36.73 ± 0.71 | 36.53 ± 0.82 | 36.31 ± 1.03 | 36.14 ± 1.16 | 36.05 ± 1.31 | 36.76 ± 1.00 | 37.35 ± 0.84 | 37.79 ± 0.73 | 38.10 ± 0.60 | 38.38 ± 0.53 | 38.60 ± 0.50 | 38.76 ± 0.49 | 38.90 ± 0.44 |
| T_b_ | CON | 35.93 ± 0.55 | 36.08 ± 0.53 | 36.08 ± 0.57 | 36.08 ± 0.61 | 36.00 ± 0.68 | 35.92 ± 0.80 | 35.92 ± 0.83 | 36.17 ± 0.96 | 36.62 ± 0.74 | 37.09 ± 0.62 | 37.45 ± 0.52 | 37.71 ± 0.45 | 37.90 ± 0.43 | 38.09 ± 0.40 | 38.19 ± 0.42 | 38.26 ± 0.42 |
|  | INT | 35.90 ± 0.51 | 36.05 ± 0.49 | 36.01 ± 0.56 | 35.99 ± 0.56 | 35.87 ± 0.66 | 35.77 ± 0.80 | 35.77 ± 0.84 | 36.01 ± 1.01 | 36.49 ± 0.74 | 37.02 ± 0.62 | 37.42 ± 0.48 | 37.69 ± 0.38 | 37.89 ± 0.36 | 38.07 ± 0.35 | 38.20 ± 0.36 | 38.27 ± 0.37 |
|  | EXT | 35.76 ± 0.51 | 35.90 ± 0.49 | 35.86 ± 0.56 | 35.83 ± 0.54 | 35.73 ± 0.59 | 35.62 ± 0.77 | 35.63 ± 0.85 | 35.94 ± 0.84 | 36.48 ± 0.67 | 36.99 ± 0.58 | 37.37 ± 0.49 | 37.67 ± 0.40 | 37.86 ± 0.36 | 38.04 ± 0.34 | 38.14 ± 0.34 | 38.22 ± 0.32 |
|  | MIX | 35.82 ± 0.54 | 35.95 ± 0.52 | 35.84 ± 0.65 | 35.72 ± 0.66 | 35.57 ± 0.71 | 35.36 ± 0.97 | 35.33 ± 1.05 | 35.62 ± 1.06 | 36.28 ± 0.85 | 36.89 ± 0.75 | 37.32 ± 0.66 | 37.65 ± 0.53 | 37.92 ± 0.45 | 38.11 ± 0.42 | 38.23 ± 0.40 | 38.31 ± 0.38 |
| Mean Tsk | CON | 32.46 ± 1.22 | 33.60 ± 0.97 | 34.03 ± 0.84 | 34.13 ± 1.32 | 34.27 ± 1.25 | 34.38 ± 1.24 | 34.48 ± 1.25 | 34.60 ± 1.13 | 34.82 ± 1.08 | 35.18 ± 1.06 | 35.39 ± 0.97 | 35.56 ± 0.90 | 35.73 ± 0.83 | 35.89 ± 0.74 | 35.86 ± 0.88 | 35.83 ± 0.81 |
|  | INT | 32.84 ± 0.83 | 34.04 ± 0.70 | 34.45 ± 0.66 | 34.68 ± 0.63 | 34.82 ± 0.55 | 34.96 ± 0.57 | 35.01 ± 0.49 | 35.10 ± 0.42 | 35.25 ± 0.44 | 35.46 ± 0.61 | 35.63 ± 0.74 | 35.71 ± 0.66 | 35.89 ± 0.71 | 35.99 ± 0.73 | 36.03 ± 0.75 | 35.90 ± 0.89 |
|  | EXT | 30.58 ± 1.01 | 31.14 ± 1.00 | 31.42 ± 0.97 | 31.60 ± 1.01 | 31.68 ± 0.99 | 31.75 ± 1.05 | 32.31 ± 1.18 | 33.25 ± 1.08 | 34.02 ± 0.9 | 34.72 ± 0.76 | 35.10 ± 0.72 | 35.27 ± 0.66 | 35.40 ± 0.65 | 35.43 ± 0.61 | 35.42 ± 0.70 | 35.10 ± 0.85 |
|  | MIX | 31.09 ± 1.09 | 31.67 ± 0.86 | 31.94 ± 0.62 | 32.06 ± 0.53 | 31.97 ± 0.70 | 32.01 ± 0.83 | 32.59 ± 0.93 | 33.58 ± 0.78 | 34.37 ± 0.62 | 35.04 ± 0.55 | 35.50 ± 0.47 | 35.80 ± 0.36 | 36.00 ± 0.36 | 36.08 ± 0.35 | 36.04 ± 0.37 | 35.71 ± 0.69 |

**Supplementary Table:** Mean (±SD) T_gi_, T_b_ and Mean T_sk_ measured during pre-cooling and at 5-min intervals during exercise. Data collected in all 4 conditions are presented (CON: control, INT: internal cooling, EXT: external cooling and MIX: internal and external cooling).
